# Supplementary material for: Genome-Wide Identification and Expression Analysis of Isopentenyl transferase Family Genes during Development and Resistance to Abiotic Stresses in Tea Plant (Camellia sinensis)
Source: Plants (Basel). 2022 Aug 29;11(17):2243. doi: 10.3390/plants11172243 (PMC9460862; doi:10.3390/plants11172243)
Supplement: Supplementary file 1 [file plants-11-02243-s001.zip › Figures.pdf]

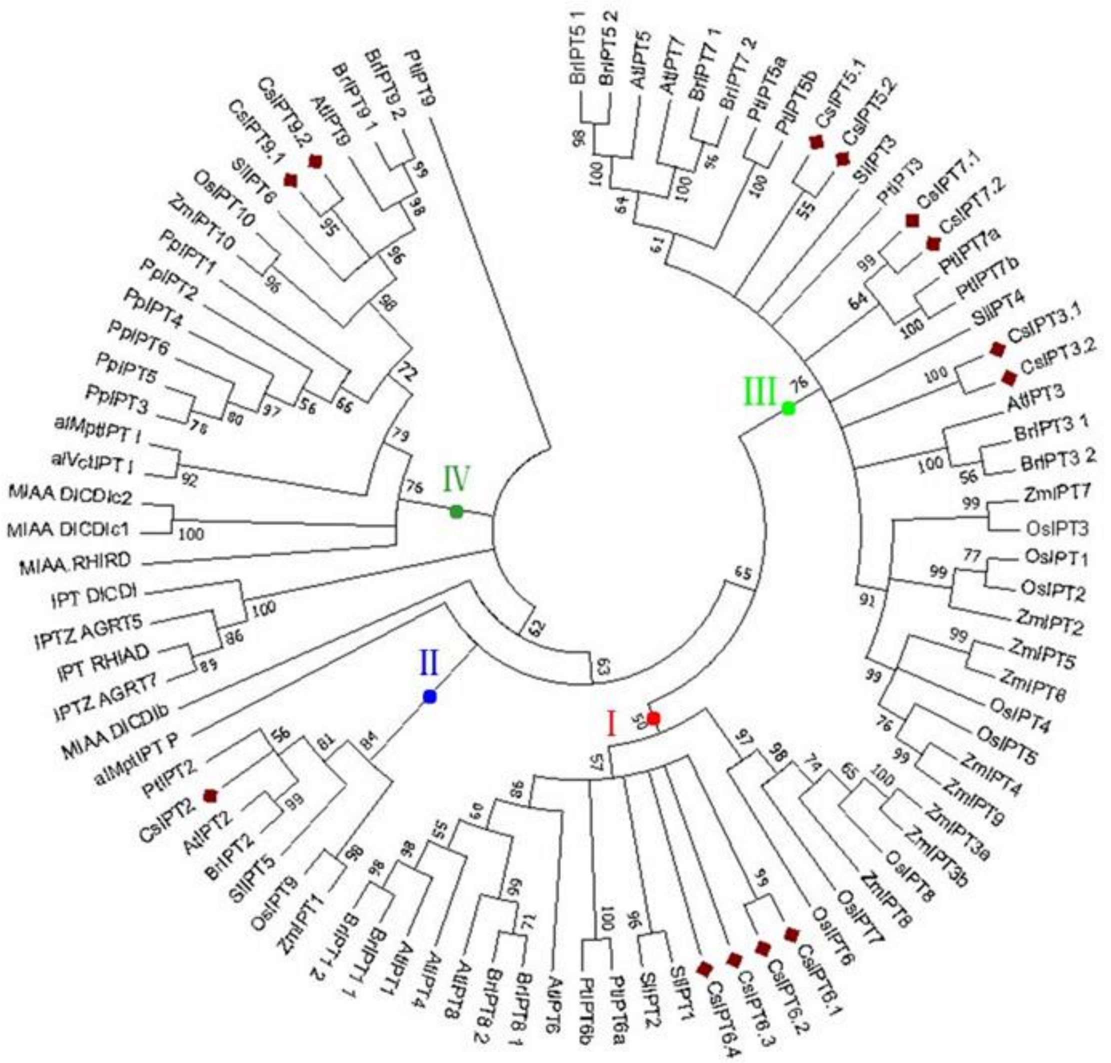

Figure S1. Phylogenetic relationships of IPT gene family from several species. Phylogenetic relationships of *IPT* gene family from several species. Trees were constructed using the NJ method by the MEGA program. The phylogenetic tree of *IPT* gene family contained two kinds of bacteria *Agrobacterium tumefacien* (*IPTZ AGRT5*, *IPTZ AGRT7*, *MIAA.RHIRD*, and *IPT RHIAD*) and *Dictyostelium discoideum* (*IPT DICDI*, *MIAA DICDIb*, *MIAA DICDIc1*, and *MIAA DICDIc2*); two kinds of algaes *Volvox carteri* (*alVctIPT I*) and *Micromonaspusilla* (*alMptIPT I* and *alMptIPT P*); *Physcomitrella patens* (*PpIPT1-6*); two kinds of monocots *Zea mays* (5 *ZmIPT*genes) and *Oryza sativa* (*OsIPT1-10*); five kinds of dicots *Arabidopsis thaliana* (*AtIPT1-9*), *Brassica rapa* (13 *BrIPT* genes), *Populus trichocarpa* (9 *PtIPT* genes), *Solanum lycopersicum* (*SlIPT1-6*) and *Camellia sinensis* (13 *CsIPT*genes). The *IPT* genes were divided into four groups indicated by differently colored dots. *CsIPT* genes were highlighted by brown rectangles.

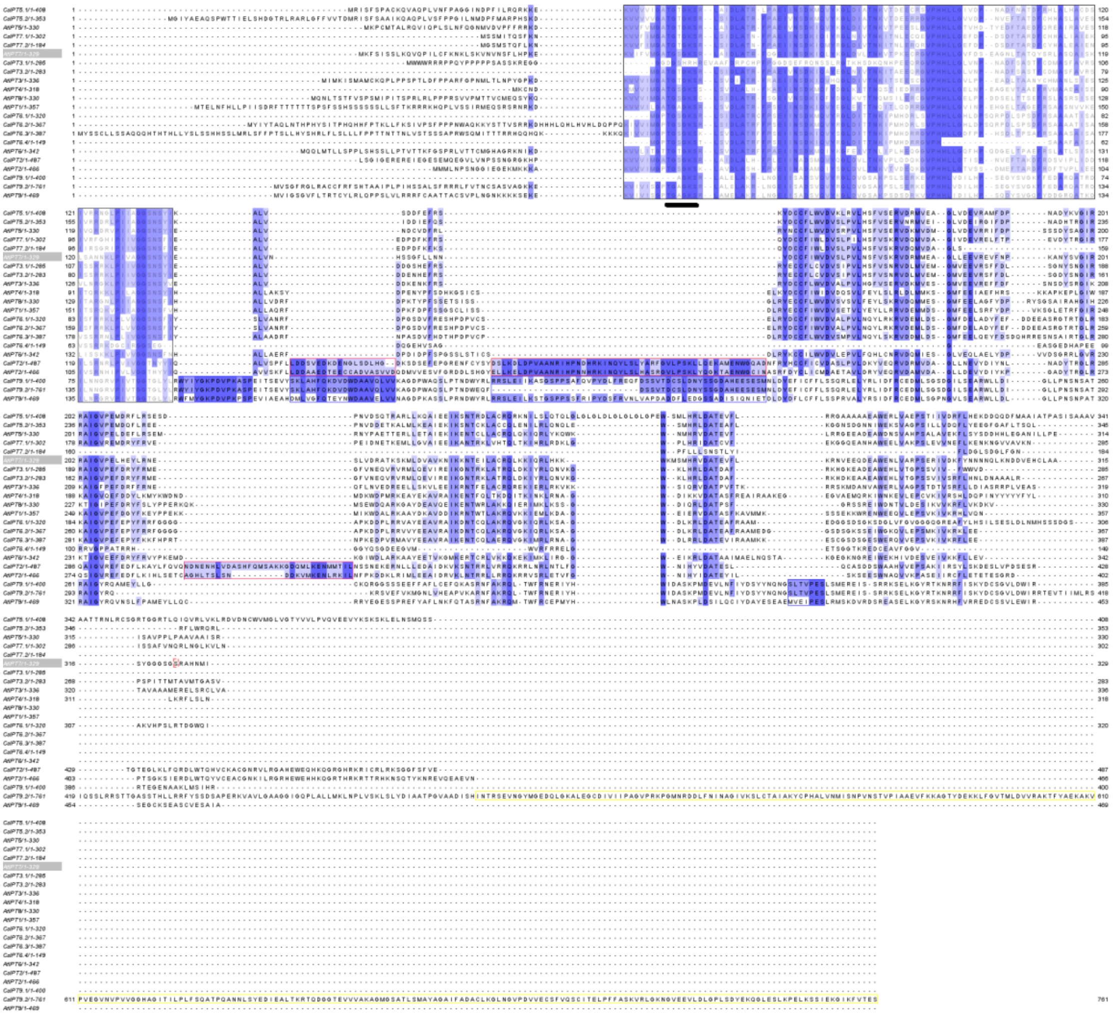

Figure S2. The multiple sequence alignment of tea plant CsIPT sequences with Arabidopsis AtIPTs.
