# Supplementary material for: Genome-Wide Identification and Expression Analysis of Isopentenyl transferase Family Genes during Development and Resistance to Abiotic Stresses in Tea Plant (Camellia sinensis)
Source: Plants (Basel). 2022 Aug 29;11(17):2243. doi: 10.3390/plants11172243 (PMC9460862; doi:10.3390/plants11172243)
Supplement: Supplementary file 1 [file plants-11-02243-s001.zip › Table S1.pdf]

Table S1 The amino acid sequence of IPT numbers from several species

>AtIPT1

MTELNFHLLPIISDRFTTTTTTSPSFSSHSSSSSSLLSFTKRRRKHQPLVSSIRMEQSRSRNRK
DKVVVILGATGAGKSRLSVDLATRFPSEIINSDKIQVYEGLEITTNQITLQDRRGVPHHLLG
VINPEHGELTAGEFRSAASNVVKEITSRQKVPIIAGGSNSFVHALLAQRDPKFDPFSSGSCLI
SSDLRYECCFIWVDVSETVLYEYLLRRVDEMMDSGMFEELSRFYDPVKSGLETRFGIRKAI
GVPEFDGYFKEYPPEKKMIKWDALRKAAYDKAVDDIKRNTWTLAKRQVKKIEMLKDAG
WEIERVDATASFKAVMMKSSSEKKWRENWEEQVLEPSVKIVKRHLVQN

>AtIPT2

MMMLNPSNGGIEGEKMKKKAKVVVIMGPTGSGKSKLAVDLASHFPVEIINADAMQIYSG
LDVLTNKVTVDEQKGVPHHLLGTVSSDMEFTARDFRDFTVPLIEEIVSRNHIPVLVGGTHY
YIQAVVSKFLLDDAAEDTEECCADVASVVDQDMVVESVFGRDDLSHGYELLKELDPVAA
NRIHPNNHRKINQYLSLHASRGVLPSKLYQGKTAENWGCINASRFDYCLICMDAETAVLD
RYVEQRVDAMVDAGLLDEVYDIYKPGADYTRGLRQSIGVREFEDFLKIHLSETCAGHLTS
LSNDDKVMKENLRKILNFPKDDKLRIMLEEAIDRVKLNTRRLLRRQKRRVSRLETVFGWN
IHYIDATEYILSKSEESWNAQVVKPASEIIRCFLETETESGRDPTSGKSIERDLWTQYVCEAC
GNKILRGRHEWEHHKQGRTHRKRTTRHKNSQTYKNREVQEAEVN

>AtIPT3

MIMKISMAMCKQPLPPSPTLDFPPARFGPNMLTLNPYGPKDKVVVIMGATGTGKSRLSVDI
ATRFRAEIINSDKIQVHQGLDIVTNKITSEESCGVPHHLLGVLPPEADLTAANYCHMANLSI
ESVLNRGKLPIIVGGSNSYVEALVDDKENKFRSRYDCCFLWVDVALPVLHGFVSERVDKM
VESGMVEEVREFFDFSNSDYSRGIKKAIGFPEFDRFFRNEQFLNVEDREELLSKVLEEIKRN
TFELACRQREKIERLRKVKKWSIQRVDATPVFTKRRSKMDANVAWERLVAGPSTDTVSRF
LLDIASRRPLVEASTAVAAAMERELSRCLVA

>AtIPT4

MKCNDKMVVIMGATGSGKSSLSVDLALHFKAEIINSDKMQFYDGLKITTNQSTIEDRRGV
PHHLLGELNPEAGEVTAAEFRVMAAEAISEITQRKKLPILAGGSNSYIHALLAKSYDPENY
PFSDHKGSICSELKYDCCFIWIDVDQSVLFEYLSLRLDLMMKSGMFEEIAEFHRSKKAPKE
PLGIWKAIGVQEFDDYLKMYKWDNDMDKWDPMRKEAYEKAVRAIKENTFQLTKDQITK
INKLRNAGWDIKKVDATASFREAIRAAKEGEGVAEMQRKIWNKEVLEPCVKIVRSHLDQP
INYYYYYFYLLKRFLSLN

>AtIPT5

MKPCMTALRQVIQPLSLNFQGNMVDVPFFRRKDKVVFVMGATGTGKSRLAIDLATRFPA
EIVNSDKIQVYKGLDIVTNKVTPEESLGVPHHLLGTVHDTYEDFTAEDFQREAIRAVESIV
QRDRVPIIAGGSNSYIEALVNDCVDFRLRYNCCFLWVDVSRPVLHSFVSERVDKMVDMGL
VDEVRRIFDPSSSDYSAGIRRAIGVPELDEFLRSEMRNYPAETTERLLETAIEKIKENTCLLA
CRQLQKIQRLYKQWKWNMHRVDATEVFLRRGEEADEAWDNSVAHPSALAVEKFLSYSD
DHHLEGANILLPEISAVPPLPAAVAAISR

>AtIPT6

MQQLMTLLSPPLSHSSLLPTVTTKFGSPRLVTTCMGHAGRKNIKDKVVLITGTTGTGKSRL
SVDLATRFFPAEIINSDKMQIYKGFEIVTNLIPLHEQGGVPHHLLGQFHPQDGELTPAEFRSL
ATLSISKLISSKKLPIVVGGSNSFNHALLAERFDPDIDPFSPGSSLSTICSDLRYKCCILWVDV
LEPVLFQHLCNRVDQMIESGLVEQLAELYDPVVDSGRRLGVRKTIGVEEFDRYFRVYPKE
MDKGIWDLARKAAYEETVKGMKERTCRLVKKQKEKIMKLIRGGWEIKRLDATAAIMAEL

NQSTAKGEGKNGREIWEKHIVDESVEIVKKFLLEV
>AtIPT7
MKFSISSLKQVQPILCFKNKLSKVNVNSFLHPKEKVIFVMGATGSGKSRLAIDLATRFQGEI
INSDKIQLYKGLDVLTNKVTPKECRGVPHHLLGVFDSEAGNLTATQYSRLASQAISKLSAN
NKLPIVAGGSNSYIEALVNHSSGFLLNNYDCCFIWVDVSLPVLNSFVSKRVDRMMEAGLL
EEVREVFNPKANYSVGIRRAIGVPELHEYLRNESLVDRATKSKMLDVAVKNIKKNTEILAC
RQLKKIQRLHKKWKMSMHRVDATEVFLKRNVEEQDEAWENLVARPSERIVDKFYNNNN
QLKNDDVEHCLAASYGGGSGSRAHNMI
>AtIPT8
MQNLTSTFVSPSMIPITSPRLRLPPPRSVVPMTTVCMEQSYKQKVVVIMGATGSGKSCLSID
LATRFSGEIVNSDKIQFYDGLKVTTNQMSILERCGVPHHLLGELPPDDSELTTSEFRSLASR
SISEITARGNLPIIAGGSNSFIHALLVDRFDPKTYPFSSETSISSGLRYECCFLWVDVSVSVLF
EYLSKRVDQMMESGMFEELAGFYDPRYSGSAIRAHGIHKTIGIPEFDRYFSLYPPERKQKM
SEWDQARKGAYDEAVQEIKENTWRLAKKQIERIMKLKSSGWDIQRLDATPSFGRSSREIW
DNTVLDESIKVVKRFLVKDKV
>AtIPT9
MVIGSGVFLTRTCYLRLQPPSLVLRRRFCAATTACSVPLNGNKKKKSEKEKVIVISGPTGA
GKSRLAMELAKRLNGEIISADSVQVYKGLDVGSAKPSDSDRKVVPHHLIDILHPSQDYSV
GQFYDDGRQATKDILNRGRVPIVTGGTGLYLRWFMYGKPDVPKPSPEVIAEAHDMLVGF
QTEYNWDAAVELVVNAGDPKASSLPRNDWYRLRRSLEILKSTGSPPSSFRIPYDSFRVNLV
APDADDFLEDGSSADISIQNIETDLDYDFLCFFLSSPRVALYRSIDFRCEDMLSGPNGVLSEA
RWLLDLGLLPNSNPATRAIGYRQVNSLFPAMEYLLQCRRYEGESSPREFYAFLNKFQTASR
NFAKRQMTWFRCEPMYHWLNASKPLDSILQCIYDAYESEAEMVEIPESLRMSKDVRDSR
EASELKGYRSKNRHFVRREDCSSVLEWIRSEGCKSEASCVESAIA
>CsIPT2
LSGIGEREREIEGESEMQEGVLVNPSSNGRGKHPKVVVIMGATGSGKSRLAIDLALHFPLEI
INADSMQVYQGLDVLTNKVPLQDQKGVPHHLLGTISPNVEFTAKDFRDSVIPLIDDILSRN
RLPVIVGGTNYYIQALVSPFLLDDSVEDKDENGLSDLHGDKSDSEFEPGRENFCYSYDSLK
DLDPVAANRIHPNDHRKINQYLSLYARFGVLPSKLLQEKAMENWGQADNFRYHCCFICVD
ASLPVLDKYVEQRVDRMVDAGLLNEVYDIYNLNADYTRGLRQAIGVREFEDFLKAYLFQ
VQNDNENHLVDASHFQMSAKKGDQMLKENMMTILNSSNEKERNLLLEDAIDKVKSNTR
RLVRRQKRRLNRLNTLFGWNIHYVDATESLQCASDDSWAAHVVEPSAKIIRSYLSKDESL
VPDSESRTGTEGLKLFQRDLWTQHVCKACGNRVLRGAHEWEQHKQGRGHRKRICRLRK
SGGFSFVE
>CsIPT3.1
MWWWRRRPPQYPPPPPSASSKREGGGGDGSHRHREVAAFDRPCHPFPGGDSEFRQNSSL
RGTKHSDKQNHPEEQRGVPHHLLGLVNPNADFSASTFCDMASFAVRSISSRRKLPIIVGGS
NSYIEALVDDGSHEFRSRYECCFLCVDVSIPVLHSFVSNRVDLMVEMGMVEEVRSFFDLS
GNYSNGIRRAIGVPEFDRYFRMEGFVNEQVRVRMLQEVIREIKGNTRKLATRQLDKIYRL
QNVKGWKLHRLDATDAFRKHGKEADEAWEHLVTGPSSVIVFWWVD
>CsIPT3.2
MGATGTGKSRLSIDLATRFPAEIVNSDKIQAYEGLNIVTNKITAEEQRGVPHHLLGLVNPNA
DFSASTFCDMASFAVRSISSRRKLPIIVGGSNSYIEALVDDENHEFRSRYECCFLCVDVSIPV
LHSFVSNRVDLMVEMGMVEEVRSFFDLSGDYSNGIRRAIGVPEFDRYFRMEGFVNEQVR

VRMLQEVIREIKGNTRKLATRQLDKIYRLQNVKGWKLHRLDATDAFRKHDKEAAEAWE
HLVTGPSSVIVSRFLHNLDNAAALRPSPITTMTAVMTGASV
>CsIPT5.1
MRISFSPACKQVAQPLVNFPAGGINDPFILRQRKKEKVVVVIGATGTGKSRLAIDLATRLPA
EIINSDKMQVYKGLDIATNKVTDEECRSVPHHLLGIVDPNADFNATDFRHLALHACDSIVR
RNGLPIIAGGSNSYIKALVSDDFEFRSKYDCCFLWVDVKLRVLHSFVSERVDRMVEAGLV
DEVRAMFDPNADYKVGIRRAIGVPEMDRFLRSESDPNVDSQTRARLLKQAIEEIKSNTRD
LACRQRKNILSLQTQLGLGLGLDLGLGLGLGPEWSMLHRLDATEVFLRRGAAAAAEAWE
RLVAEPSTIIVDRFLHEKDDQQDFMAAIATPASISAAAVAATTRNLRCSGRTGGRTLQIQVRL
VKLRDVDNCWVMGLVGTYVVLPVQVEEVYKSKSKLELNSMQSS
>CsIPT5.2
MGIYAEAQSPWTTIELSHDGTRLRARLGFFVVTDMRISFSAAIKQAQPLVSFPPGILNMDPF
MARPHSKDKVVVVVVGATGTGKSRLSIDLATRFPAEIVNSDKMQVYKGLDIATNKVTDEER
RGVPHHLLGVVDPNVEFTATDFCHHASLAVESIVRRDRLPIIAGGSNSYIKALVIDDIEFQSK
YDCCFLWVDVSLPVLHSFVSERVNRMVEIGLVDEIRGIFDPNADHTRGIRRAIGVPEMDQF
LREEPNVDDETKALMLKEAIEKIKSNTCKLACCQLENILRLQNQLEWSMHRLDATEAFLK
GGNSDGNNIWEKSVAGPSILLVDQFLYEEGFGAFLTSQLRFLWRQRL
>CsIPT6.1
MGPTGSGKSRLSIDLATRCFPSEIINSDKMQVYRGLDITTNKIPMHDRLSVPHHLLGHLDPS
QRPDLSPSDFRSAAAATISAISFRRKLPLVVGGSNSFIYSLVANRFDPGSDVFRESHPDPVCS
ELRYDCCFIWVDVALPVLNQYLRKRVDEMLDSGMFDELADYFEFDDEEASRGTRTGLRK
AIGVPEFEPYFRRFGGGGAPKDDPLRRVAYEEAVRAIKDNTCQLAKRQVGKIQRLKSAGW
DLKRLDATEAFRAAMEDGGSDSGKSDGLVFGVGGGQGREAFYLHSILSESLDLNMHSSS
DGSAKVHPSLRTDGWQI
>CsIPT6.2
MYIYTAQLNTHPHYSITPHQHHFPTKLLFKSIVPSFPPPNWAQKKYSTTVSRRKDHHHLQH
LHVHLDQPPQKIVVIMGPTGSGKSRLSIDLATRFFPSEIINSDKMQVYRGLDITTNKIPMHD
RLSVPHHLLGHLDPSQRPDLSPSDFRSAAAATISAISFRRKLPLVVGGSNSFIYSLVANRFDP
GSDVFRESHPDPVCSELRYDCCFIWVDVALPVLNQYLRKRVDEMLDSGMFDELADYFEF
DDEEASRGTRTGLRKAIGVPEFEPYFRRFGGGGAPKDDPLRRVVYEEAVRAIKDNTCQLA
KRQVGKIQRLKSAGWDLKRLDATEAFRAAMEDGGSDSGKSSEIWGKQVLEPSMKIVKRK
LEE
>CsIPT6.3
MYSSCLLSSAQQQHTHTHLLYSLSSHHSSLMRLSFFPTSLLHYSHRLFLSLLLFPPTTNTTN
LVSTSSSAPRWSQMITTTRRHQQHQKKKKQKIVVIMGPTGSGKSKLSVDLATRFFPSEIINS
DKIQVYSGLDITTNKIPMHDRRGVPHHLLGDFPPHSDLTPSAFRSAAASAISAVSSRRNLPL
IVGGSNSFIYALAADRFNPASDVFHDTHPDPVCPDLRYDCCFLWVDVSMPVLNQYLSKRV
DDMLDSGMFEELQEYFDSDQHRRESNSAIRTGLRKAIGVPEFEPYFKKFHPRTNPKEDPVR
MAVYEEAVRAIKENTCQLAERQVGKIMRLRNAGWDLRRLDATEVIRAAMKKESCGSDSG
ESWERQVVEPSVKIVKRFLEE
>CsIPT6.4
MGPTGSGKSTLSVDLATRFFPSEIINSDKIQVYRGLDITTNKIPMHDRRGVPHSAAAFAISA
VSSRRKDGCIRGGSEGDQGEHVSAGGEASGEDHAPEERRVGPPATRRHGGYQSGDEEGV
MWVRFRRELGETSGGTKREDCEAVFGGV

>CsIPT7.1
MSSMITQSFKNKVVFIMGATGTGKSRLSIDLATNFPAEIINSDKIQIYRGLNIVTNKITNLEC
QEVPHHLLGEFDPDSDFTARDFCHHALIAIEKIVRFGHIPIIVGGSNSFIEALVEDPDFKFRSQ
YDCCFIWLDVSLPILHSFVSKRVDQMVDAGIVDEVRELFTPEVDYTRGIRRAIGVREMDR
YFRVEPEIDNETKEMLLGVAIEEIKANTRKLVHTQLTKIHRLRDKLGWPLHRIDATCVFEK
GGQEANHAWEELVAKPSLEVVNEFLKENKNGVVAVKNISSAFVNQRLNGLKVLN
>CsIPT7.2
MGSMSTQFLKNKVVFIMGATGTGKSRLSIDLATNFPAEIINSDKIQMYRGLDIVTNKITNLE
RQRVPHHLLGEFDPDSDFTARDFCYHALEAIENIIRSGRIPIIVGGSNSFIEALVEDPDFKFKS
QYDCCFIWLDVSLPVLHSFVSKRVDQMVDAGLSWPFLLLSNSTLYIFLDGLSDGLFGN
>CsIPT9.1
AGKSRLALELAKRLNGEIISADSVQVYRGLDVGSAKPSLSERKEVPHHLVDILHPSEGYSV
GKFFEDARQATRDILNNGRVPIVTGGTGLYLRWYIYGKPDVPKASPEITSEVYSKLAHFQK
DVDWDAAVQLVVKAGDPWAQSLPTNDWYRLRRSLEIIKASGSPPSAFQVPYDLFREQFDS
SVTDCSLDNYSSGDAHEESESMNLDYEFICFFLSSQRLELYRLIDFRCEDMLSGSDGILSEA
RWLLDIGLLPNSNSATRAIGYRQAMEYLLGCKQRGGSSSEEFFAFLCEFQKASRNFAKRQL
TWFRNERIYHWIDASKPMDEVLNFIYDSYYNQNGSLTVPESLSMEREISSRRKSELKGYRT
KNRRFISKYDCSGVLDWIRRTEGENAAKLMSIHR
>CsIPT9.2
MVSGFRGLRACCFRFSHTAAIPLPIHSSALSFRRRLFVTNCSASVAGKKEKVIVISGPTGAG
KSRLALELAKRLNGEIISADSVQVYRGLDVGSAKPSLSERKEVPHHLVDILHPSEGYSVGK
FFEDARQATRDILNNGRVPIVTGGTGLYLRWYIYGKPDVPKASPEITSEVYSKLAHFQKDV
DWDAAVQLVVKAGDPWAQSLPTNDWEQFDSSVTDCSLDNYSSGDAHEESESMNLDYEFI
CFFLSSQRLELYRLIDFRCEDMLSGSDGILSEARWLLDIGLLPNSNSATRAIGYRQKRSVEF
VKMGNLVHEAPVKARNFAKRQLTWFRNERIYHWIDASKPMDEVLNFIYDSYYNQNGSLT
VPESLSMEREISSRRKSELKGYRTKNRRFISKYDCSGVLDWIRRTEVTIIMLRSIQSSLRRST
TGASSTHLLRRFYSSDSAPERKVAVLGAAGGIGQPLALLMKLNPLVSKLSLYDIAATPGVA
ADISHINTRSEVNGYMGEDQLGKALEGCDIVIIPAGVPRKPGMNRDDLFNINAGIVKSLCT
AIAKYCPHALVNMISNPVNSTVPIAAEVFKKAGTYDEKKLFGVTMLDVVRAKTFYAEKA
KVPVEGVNVPVVGGHAGITILPLFSQATPQANNLSYEDIEALTKRTQDGGTEVVVAKAGM
GSATLSMAYAGAIFADACLKGLNGVPDVVECSFVQSCITELPFFASKVRLGKNGVEEVLDL
GPLSDYEKQGLESLKPELKSSIEKGIKFVTES
>IPTZ_AGRT5
MLLHLIYGPTCSGKTDMAIQIAQETGWPVVALDRVQCCPQIATGSGRPLESELQSTRRIYL
DSRPLTEGILDAESAHRRLIFEVDWRKSEEGLILEGGSISLLNCMAKSPFWRSGFQWHVKR
LRLGDSDAFLTRAKQRVAEMFAIREDRPSLLEELAELWNYPAARPILEDIDGYRCAIRFARK
HDLAISQLPNIDAGRHVELIEAIANEYLEHALSQERDFPQWPEDGAGQPVCPVTLTRIR
>IPTZ_AGRT7
MDLRLIFGPTCTGKTSTAVALAQQTGLPVLSLDRVQCCPQLSTGSGRPTVEELKGTSRLYL
DDRPLVKGIIAAKQAHERLMGEVYNYEAHGGLILEGGSISLLKCMAQSSYWSADFRWHII
RHELADEETFMNVAKARVKQMLRPAAGLSIIQELVDLWKEPRLRPILKEIDGYRYAMLFAS
QNQITSDMLLQLDADMEDKLIHGIAQEYLIHARRQEQKFPRVNAAAYDGFEGHPFGMY
>MIAA.RHIRD
MMKNLDQNFDAILITGPTASGKSALALRLARERNGVVINADSMQVYDTLRVLTARPSDHE

MEGVPHRLYGHVPAGSAYSTGEWLRDISGLLSDLRGEGRFPVIVGGTGLYFKALTGGLSD
MPAIPDDLREGLRARLIEEGAAKLHAELVSRDPSMAQMLQPGDGQRIVRALEVLEATGKSI
RDFQRASGPMIIDPERAQKFIVLPERPVLHDRINRRFEAMMDSGAVEEVQALLALNLAPDA
TAMKAIGVAQIADMLTGRMGAAEVIEKSAAATRQYAKRQMTWFRNQMGDDWTRIQP
>IPT_RHIAD
MDLRLIFGPTCTGKTSTAIALAQQTGQPVLSLDRVQCCPQLSTGSGRPTVEELKGTTRLYL
DEQPLVKGIISAKQAHERLIAEVYNNEAHGGLILEGGSISLLKCMVQSSYWSNDFRWRIIR
HKLADEETFMKAAKARVKQMLHPAAGLSIIEELVHLWNQPQLRPILEGIDGYRYAMLFAS
QNQITPDMLLQLGADMEDKLAHGIAQEYLIHARRQEQEFPSINAVAFEGFEGHPFGM
>IPT_DICDI
MKILLIIGSTGVGKTDLSINYSKKYNAPVVVLDRIQCFPELSITSGRPDESEYFGSKRIYLTD
LLVEPGNENIKKTFYVNKLINILNEIKNNYDTQNLPNEKGYGCIFEGGSISLLKELLTKINKL
PYKITCVIYIRPSDSIDNHKLYKAKIFKRVSQMLFPTEEGNDSQILEVKRILNKGKTVNAQG
EINLEYYEKIKQVLISLVGLVGIEDVIHFLDKQYDQKNITSKLDPNYLNEIQSQLIETITLAH
YNYALSQIELIDSLIKQLPKSIEYCLKNIEIN
>MIAA_DICDIb
MTSPLVVFVLGTTGVGKSKLAIELAKQFNGEIISSDSMQLYKDGGDITTNKVTIEEMEGIPH
HMMSFLPIDTLNYNVADFASKALGIIRDILSRGKLPIVVGGTHYYTCSLLWSDSIISLDDND
NNNNNNNNNNNNNNNNNNNNNNNNNNNNNNNNNNNNDNNSENKIILEEEEEEIIDKNK
YSYEILKEIDPIMAEKLHKNDIRKIKRSLDIYYTSGKKQSDLINDQHDKKSLRFRSCLLWLE
CKDQSILEYRLNKRVDNMIELGMLDEVFNIFKILNKSNTENFSKGLTQAIGIKELYDYYLFL
NENENTKIENENKEIDNDNENNNNNNNNNKIKKIEKLKIKKEKQREKLYKESIDSIKSHTK
RYAIRQIKWISKISPINTPLDILSLDSSNLDLWNDLVFIPSKNKVDQFINHLNLTGYKNKDST
NNDNNNINNNINNNNNNNNNDLNKWKKYYCEHCSKELNGDNEWSFHQKSKQHRHRLKK
RDTNNIDNKDKYNNNNKNNNNNNIQKEKGEIETEK
>MIAA_DICDIc1
MKRFFGSGGIKMKDKVIIIAGGTGIGKSDLSLKLAKQINGEIIGADSVQIYKHLTIASNKVT
DTNGIPHHLIDMLDLDDNNFCLFDYYRLAKNTIKDVLSRGRVPIVVGGCGFYLDTILKGSR
VDLTNDKERDEIKLYYNKIKQEDNWDYYYDLLKKYDIESANMILKNDFIRLSKSLYFNTV
KGVKFSTQKDDYQDSIANEYDFRTFYLSGDRSKMHITLNNRCESMFKMGILEEVYDLLKR
DKDLHFKNSKSSESIGYRQIMDLLLKPVVRNIDDTSINMNQSLNAIDESDVISTILNFQTAN
RNYYRKQYSWFKKEKYFEWLNPIDNEITNQYIIKSLELPFDEWQNNRNLKKEEEIRTCSPK
DTKLISLYKPSKQLVSSILLNSNKLRNSLIKSNPELFENDNNNNKYK
>MIAA_DICDIc2
MKRFFGSGGIKMKDKVIIIAGGTGIGKSDLSLKLAKQINGEIIGADSVQIYKHLTIASNKVT
DTNGIPHHLIDMLDLDDNNFCLFDYYRLAKNTIKDVLSRGRVPIVVGGCGFYLDTILKGSR
VDLTNDKERDEIKLYYNKIKQEDNWDYYYDLLKKYDIESANMILKNDFIRLSKSLYFNTV
KGVKFSTQKDDYQDSIANEYDFRTFYLSGDRSKMHITLNNRCESMFKMGILEEVYDLLKR
DKDLHFKNSKSSESIGYRQIMDLLLKPVVRNIDDTSINMNQSLNAIDESDVISTILNFQTAN
RNYYRKQYSWFKKEKYFEWLNPIDNEITNQYIIKSLELPFDEWQNNRNLKKEEEIRTCSPK
DTKLISLYKPSKQLVSSILLNSNKLRNSLIKSNPELFENDNNNNKYK
>alVctIPT_I
MPATSLFELQLQPQYAPQQQQQQQQPSLSPPPPPPPPQQQQQQHTQDEHEQEAARAAAAT
ADNVPAEGSETAQRRPMVVIVTGPTAVGKTAISLMLATHLRQPTTTGHHNYQHQEQQQEQ

QQEQQQHQQEQQQEQRHSDGNGAGYSATGIGCGAVDGAGGCSAAGGGGASSAGGEVIS
ADSVQVYKGLDVGSDKLPPSERRGIPHHLIDVREPHQDFSAGEFFECARPVMEDIINRGGV
PLVVGGTGLYLRWLVVGKGGAPRATPEALKAVNEAIGAAWAEREAEKRAAAVAEATDAT
AAAAAAAAGTAEVAAVAPDPFCLGEEEKWDAAAALLLKWGDDVAYDRIRAERNNYYRL
ERALAILAMHPGAKLADFEPRAILDRELDLDFRCFFLHRPRLQLYDRIASRVEAMVVEGGL
LEEAAMLLRMGLAPGANMAAKAIGYRQAMEWLMAAKAADRPVGLPDLCALHAAICQA
THKLVRAQMTWFRDDPMYKWIDVEGRDPRDVVEEILQELAKPQHEAAAEARGPAKKAK
SKSALAAEAAAAAAAAAAEAQLAPLLARINGELLPGIPEAAVAAGAASATATSAAVSGGVR
QEVGEVEE REAKRCRSGE
>alMptIPT_I
RVLVLAGPTAVGKTSLSLDLASKLDGEIISADSVQVYRGLDVGSGKLAASERRGIAHHALD
VLDPSEDFNAGAFVDLALETVEDVVSRGKVPIVVGGTGMYLRWFVGGRPATPASTPESSR
AAKEEVRAAAAAAGWDGALKLLRDAGDPDTADRVATNDWYRVERALEILRASGRPLGS
YAPESPPPFDFRCVLLDAPRVQLYRRIDDRVECMVCDGMLDEAAMMMRRGIAPGSTSAA
SAIGYRQAMEFLAARAAAAAGGGGGGATTEEDLLAFVEKTQRATRAFAKRQLTWFRGEP
EGRYRWISASDGEEAAAREAMDAFERD
>alMptIPT_P
MPSPTPRPAASPLPGVVCVLGQTGAGKSRLGAELARAIDGEVVNADAMQLHDALPIATAR
TTSEQTLGVPHHLFGVVPVPVPTRGGESSASARDERGGGGYGVTVRDYRAMALPVVEGII
ARGKVPVLVGGSDYYARAIVSRSLLDDQLGAGREGPGAEARGEDDGDARDDGASASEEE
EDGDDDDDDDNVADAPPPSRVKPNENDAAAALARHARLREVDPVSAAKLHPNNARKVR
RYLEIYDAFGEPASVVFARGKAAAAAADDDDDDESPSDTSARYRSLCLVLRADASELEPA
LARRVDAMVDAGLVAELEAAAAANVGGGVGVSQSIGFHEWVEYLRARAATPQRDVDAA
IDTMKADAIEAMKADTRRLARAQLRRCKRLERTFGWRPTYLDATATHAGARVGDAARA
DAAWREDVFEPALRAARAFVAGEDDGGGGGGGGVAEEPAWEERRCDACERTLRGDAEW
RAHVSGRRHRKRVASLRKKREGKHGVPPREKGCSDAG
> PpIPT1
MVSLQFEVGPMYMLTHRAQACLCNCTGGLKLFRGVSSLPNGGICLPKFFSYQCANWGTI
GWNTSSVGDSSRDRVLCSKVGPRITLCDSYCAPNSKLGRVRSDGSLNNFVRAFVGETIAK
TAGAVGSGKESAMAEDNVEDTDVRGRKGRVVIIAGPTGVGKSRLALALAKRLRGEIISAD
SVQVYRTLDVGSAKTPVDEREGIPHHLIDIVDPSQDYTAGYFYDDARAATELVLTKGSVPI
VVGGTGMYLRWYMTGKVGAPKATKEVFVAVEEEVDRLIKEGGGWDDALRMLADAGDL
TTGPSLARNDWYRLRRALEIIKTSGQPRSAFSKLRNCDPDAATEGASWDYDFHCYFLYQP
RSELYQKVDLRCEQMAQEGLLDEASSLLDSGLQPNSNSASRSIGYQLAMEFLMECRQVE
GAATEERFFLFLEEFQRHSRNYVKRQLTWFRNKGQSEQMFNWIDATQPLEVMVDALAKE
YERPNEVVSDVLKAASVVTKESSYKEENLLKRYRTQNRIFTSNSEALKRTLQWIRDTQCL
WRNSSTVDDLQKRMESSLTTSM
> PpIPT2
MSTTAVVHLHSAASAVTPLWSGAREPSAPEPAIVRPTVRCFNRSSGAARASFTGVDLGKCR
AWRRGLTTPFRCVATPERRASSVGKAVSQDASRLQFPFSSLDYPDFNRTAFVEPDVSVRHH
GDRSVVEKNPPSTKLVQAPRRSVKPRVILIAGPTAVGKTRLSIELAKTLGGEVINADSVQV
YQGLDVGAAKASLEERQGIPHHVMDLVPPTVEYSYEQFLRDARSATEHILAKGLVPIVVG
GTGMYMRWFMQNRAGITSHPFEDEDLTGEFDARAEDDWDYDFQCYFLYQHRADLYPRV
DIRCEQMIPALLEEAAWLLNLGVRPQTNSASKAIGYEEAMELLLEARQADGVISQERFLKF

VALFQHNSKGLVRKQIAWFRTHSRSEVRKFRWLQAGGPVEEMMEALVEEYARAPGQPTLI
ASGEELKTRSMKEQKRMKSYKPTLKIYSDAAAVAGCLAWIKSTQGPILSPARASGQCRQIL
ARLLGVGTAAVDSRRLAETAPVSGRFLKLGMCFTTLSRRFGGSFGKVQTSRLRNCVASGR
RDGGRQWNTHRCHILLQGRPVIATVLGMVPCSLEPLPQEVGIHVTGLHRLQFPSSNSVTH
VLNVWDFLYPTLEALCQCIREWIDDRMSTAEYICTFPSCSERGFSVTERRGDLKVFRSYNQ
RRCDQGNHFFSELYNRALYTQITGVINNFISGIHTYISRRRRSVSRPLVDSFVGALQESHQK
KGQVHVLVREQILFTAMIHVRQVAMHRVIIRKSDIKDALEPCGSGPEGPDTRHRVSTSTKR
TQYRCRPCDSVLESSNQSNEADDLLNNLPILNLPILTSMLRPLALASDAGGCNHLVLRAAA
FPTIPQHMFSGQERVIGVMTSSLRWFAESHCSGKRSTENKLRTS
>PpIPT3
MSSTAAAAVVASSISSDSARHEQHELQQKPQAQRCEVQTAQRIASQTRDRDSTGLAPVAA
SPAAAVPVLAAAHESGREGQGMPPLQRKWGVRGEPDKGKRDAGARHTVTYATAQESDR
GVDRGGLRDWVPKKKYSATCGLQEHHWEDRFSSVERGGAERMELVVVRAKVAVGENG
WSARLLTEAGGGASKLACLKFAGTLDKVDSRSSGKVTGLGTQKGRVVIIAGPTAVGKSRV
AIDLAKQLGGEIISADSIQVYQGLNVGSAKTPLHEREGIPHHLVDIVPPTEEYSASQFCKDA
RDATKLVLDRGRVPIVVGGTCSYLRWYMNGKTGASYPSSQQVNAAIEGKIRRLVSHSRG
WEFAVHMLAQAGDPDTAYNLARNDWHRFGQALQAILVPIFSSPLYQSQFSWIRKHQPPTG
GTDLSVKIQVSGQSRGVFPSPHNYKLDHTDGSEWDCNYDFQCYFLYQQRAELYRWIDLR
CEHMLVDPRGLLQEAAWLLDIGVMPDTSSASRGIGYQEVGFTYHPDHSSIRAMEFLVDCR
RTGGNTTEERFLAFLSGFQHISRSLVKKQLKWFRSDQHSDVKQFHWIDASQPTDQILEALR
KEYWRRPGYPSELKGSDAMKEASYKEGKTLKHYKAKNRIFTDSTVISPVLRESCAKVTRL
TCQPPIGRSWPAGGWQAKEPVWPPCTKQNLHPCSDKTVSMQDTIQNKWNHAVDYFYDQ
MMQS
>PpIPT4
MANMIAKPKLSQYNLGPQELPISFCDCYVKLQHSFKRRRTHQSKQPRERHADRQTERAR
DICSFYLLRHPTKGFFEVRREYMWTQRAACPCLSSRRGELVVGINWQQQCKDFWDPKLV
MVDLQPNVLGMARAKVAVGNGGRQAGLLKEGGETTATTTTTRKIGCLEFAGRPDNVDRS
TTTMTGVETQKDRVIVIAGPTGVGKSRFAINLAKQLGGEIISADSIQELLVDHNVDALCEGI
PAPLWRSFHVAYSFQVLICPHFALRGIFEVAGSSCLAEHCMQLNMSFCYLLEGDSSPDSPVV
AFETFHMNIASCLTELQVYNGMNVGSAKTPLHEREGVPHHLLDIVPPTEGPYCIFLMVDSI
SRNVLLYFEVVTAKPKKYSASRFFKDARDATELVLARGRVPIVVGGTCTYMRRYMSGKA
LAPKATPEVSAVIEAEIRRLVSRPGDWEMAALRLTKASDPANAYSFARNDWYRLRRALEV
TMRMHVSGQPRRAFPKQHNYTVDHMDGLEWDSDYDFQCYFFYQHRKELYHCIDLRCEQ
MIANPRGLLQEASWLLDLGLTPDSSFSSRGIGYQETCVMILMANQAMEFLMECRKAGGR
STEERFLAFLSAFQHVSRSLVKKQLAWFRSNKHSDVKQFHWIDASQPTDKILEALRKEYW
RRPGYPSELSGSDDVKHASYMEGKILRYYEDFYEPIGNCNCFGVGEADTRRPEFSTECS
>PpIPT5
MWTQRAACPCLSSRSGESVAGGNGLQQCKDLWTPKLGMVRRVELRQSKKLVVRAKLAV
GDESRQTQFLKEGVETNTRNVKCLEFAGGPDNLDGSPTKAFSRGTQKGRVVVIAGPTAVG
KSRIAIALAKQLGGEIISADSIQVYKGMNVGSAKTPLIERQGVPHHLLDMVCPTEEYSASR
FLEDARAATDRVLDRGRVPIIVGGTCTYLRWFMNGNAGAPKATSEVGATIDGDIRRLIGRP
GSWETAVHRLAQAGDLTTTNSLARNPTNANMLRRAIEVVTPWGAFHNLYTCMLNHMDG
SEWDYDYDFQCYFLYQHRAELYHWIDLRCEQMLADPRGLLQEASWLLDLGLMPDSSSAS
RGIGYQEKFSNMFLEDACCDSSILIAMEFLMECRKAGGMATEKRVLEFLSAFQHVSRSLV

KKQLTWFRSDQHSDVKQFHWIDASQSTEKILEAMRKEYWRPPGYPSELRGSDDRKHASY
KEGKVLKYYKVENRIFTNSAAIANVLKWVKQTQVDRRFQQNVVRF
>PpIPT6
MAHSQSYKHTEFAPIGHKLSSIHIRNTPITEGHELQGGASLKSTGKSKSAAFSTSSLERKSM
LRESVTQHTGLLRVPSRKLRRTQSLVAAMCSSNNNNSTCAKCIQTSLSHLELFLQQNSRLR
SLVQCCHVSQSYCWYVVCDKIVKREKCERARGNKKDLQPSLRTAISVRYSRSEVDEGNNE
RKRWEEWWSCRERIGVESMVKKKVRLVVRANVTVEKSDKQVRLLTEAGGAAGTLGCLE
LSGRLDTRSNGKVTSLGTQRGRVVVIAGPTAVGKSRVAIALAKQLRGEIISADSIQVYKGM
NVGSAKTPLLEREGVPHHLLDMISPMEGTEYSASRFLKDARAATKMVLDSGHVPIVVGG
TCTYLHWYMNGEARAPKPSPKSNAANKERIERLVDRPGDWEFAVHTLAQAGDPATAYSL
CRNDWPRLRRALEGILETQTVGWQSDQCMKVQISGQSGGVSTKPHNYTLDNMDIPEWD
YDYDFQCYFLYQNRAELYRWIDLRCEQMIAEPQGLLQEASWLLDLGLLPNTSSASRGIGY
QEAMEFLVECRGTGGITTEEKFLAFLSGFQHVSRSLVKKQLRWFRSSHDSAVKQFHWIDA
SQPTDQILEALRAEYLRRPGYPSELKGSNALIDASYKEGKTLKYYDAKNRIFTSSSTIAPVL
RWVKKTQGDLRPIPRRFVTV
>ZmIPT1
MAHPSAAAAAVSSTAPAANPSSGAREEGGARSPPSPSPSQRGRAKVVIVMGATGAGKSRL
AVDLAAHFAGVEVVSADSMQLYRGLDVLTNKAPLHEQNGVPHHLLSVIDPSVEFTCRDFR
DRAVPIIQEIVDRGGLPVVVGGTNFYIQALVSPFLLDDMAEEMQGCTLRDHIDDGLTDEDE
GNGFERLKEIDPVAAQRIHPNDHRKIKRYLELYATTGALPSDLFQGEAAKKWGRPSNSRLD
CCFLWVDADLQVLDSYVNKRVDCMMDGGLLDEVCSIYDADAVYTQGLRQAIGVREFDE
FFRAYLPRKESGEGSCASLLGMHDDQLKSLLDEAVSQLKANTRRLVRRQRRRLHRLSKDF
GWNLHRVDATEAFFCATDDSWQKKVVKPCVDVVRRFLSDNSTVLPSTSASDPSSRELWT
QYVCEACGNRVLRGAHEWEQHRQGRGHRKRVQRLKQKSLRPWPSLLPQDRS
>ZmIPT2
MEHGAVAGKPKVVFVLGATATGKSKLAIALAERFNGEVINADKIQVHDGVPIITNKVTEEE
QGGVPHHLLSVRHPDADFTAEEFRREAASAVARVLSAGRLPVVAGGSNTYIEALVEGDGA
AFRAAHDLLFVWVDAEQELLEWYAALRVDEMVARGLVSEARAAFGGAGVDYNHGVRR
AIGLPEMHAYLVAEREGVAGEAELAAMLERAVREIKDNTFRLARTQAEKIRRLSTLDGWD
VRRIDVTPVFARKADGTECHELTWKKQVWEPCEEMVRAFLEPSLTAVPGVAVTEEGNAGV
VATAAPAGDVVVPTGDVVTAVADA
>ZmIPT3a
MAAAAVAGIGSNDVPRKRLRRPSSVRIMERRSRGGSDGDCSCSSSGKELEPAEPTTRLVVI
VGATGTGKTKLSIDAAVALGGEVVNADKIQLYAGLDVTTNKVPHADRRGVPHHLLGAVR
ADAGELPAPSYRALASAAAASVAARGRVPVVAGGSNSLVHALLADRLGDGDPFEADGGY
RPSLRFPCCLLWVDVDGAALDDYLDRRVDDMVREGMVEELREYFASTSPAERASHAAGL
GRAIGVPELGAHFAGRKTLAAAIDEIKVNTRVLAAAQVRKIRRMADAWGWPVRRLDATP
TIRARLAGAGCAAEAAAWERDVGGPGLAAMRQFLDCHEQQQQQRWPLVRRQCRGMAW
>ZmIPT3b
MAAAAVAGIVSNDAPRKRPRRPPSGVRMEAMSRGGGCCCSSSDKKLEAAEPATTRLVVIV
GATGTGKTKLSIDAAVALGGEVVNADKIQLYAGLDVTTNKVPHADRRGVPHHLLGAVRA
DAGELPAPSYRALAAEAAASVAARGRVPVVAGGSNSLIHALLAADSLGAGDPFKVDDGY
RPSLRFPCCLLWVDVDGAVLDEYLDRRVDDMVREGMVEELREYFASAPPASHAAGLGKA
IGVPELGAHFAGRKSLAAAIDEIKANTRVLAAAQVRKIRRMAGAWGWPVRRLDATPTVR

ARLAGAGRAAEAAAWERDVRGPGLAAMRQFLDGHEQQERWPLARRQCRGMVV
>ZmIPT4
MSLYLAPTAAAATTTTTTLPRQLLPAPSIDLRRLERRGMALPLPPAPAPPPPLVSANNRHAG
AKHKAVVVMGATGTGKSRLAVDLALRFGGEVINSDKIQLHAGLDVTTNKVTEQERAGVP
HHLLGVARPDEEFTAADFRREATRAARAITARGRLPIVAGGSNSYVEELVDGDRAAFRDRY
DCCFLWVDVQRAVLHGCVARRVDEMRARGLVDEVAAAFDPRRNDYSRGLWRAIGAPEL
DAYLRWPGPGVDGDAESEGERDRLLAAAIEDIKSNTRRLSCRQRAKIQRLAKMWGVRRV
DATEVFRRRGDEADEAWQRLVAAPCIDAVRSFLRTDDAAATVASDLAVDGVVPVFAPAPA
AAVAG
>ZmIPT5
MAAPAMAAPPPPPACFPMPTRLTMPPTSITLPDPPPLSVGGACRRVAAKHKAVVVLGATGT
GKSRLAIDLALRFGGEVINSDKIQAHAGLDVATNKVGLAERGRVPHHLLGVVHPDAEFTA
ADFRREASRAADRAAARGRVPVIAGGSNSYVEELVEGDRRAFRDRYECCFLWVDAQLPV
LHGFVARRVDDMCRRGLVDEVAAAFDPRRTDYSRGIWRAIGVPELDAYLRARGRGHGHH
HDQMLAAALHEIKANTSRLAVRQRGKIQRLERMWRVRRVDATEVFLKRGLAADEAWQR
LVAAPCIDAVRSFLLEDQQEYSSMGTAGAMLPAAVAAAAV
>ZmIPT6
MAAAPPACSPMAARLTMPPLPDAAAPLSVVGCRRVAAKHKAVVVLGATGTGKSRLAIDL
ALRFGGEVINSDKIQAYAGLDVATNKVGPAERAAVPHHLLGVVHPDAEFTAADFRREAAG
AAARVASRGRVPIIAGGSNSYVEELVEGDRRAFRERYDCCFLWVDARLPVLHGFVARRVD
EMCRRGLVDEVAAAFDPRRTDYSRGIWRAIGVPEMDAYLRAGGHGDGDGDEQEQRARM
LAAALDEIKVNTSRLALRQRGKIQRLARMWRVRRVDATEVFLKRGHAADEAWQRLVAAP
CIDAVRSFLLEEQEYSSSMVTASMFASTAAAAV
>ZmIPT7
MAGVNGATASGGDNKAKVVLVMGATATGKSKLAIDLALRFGGEVVNSDKIQVHDGLDV
VTNKVTAAERQGVPHHLIDGVAPDADYTTADFCRDAVRAVESILERGRVPIIAGGSNRYLE
ALLDGEPPAGFRGRYECCFLWVDSDLAVLDRYIGSRVDCMLEQGLVREVRAFFRHDDAD
YSRGIRRAIGVPEMDMYFRMEAAGALDGDDDDQLRVRLLAAAVNEIKANTCGLARRQL
QKIHRLHGLQGWSDIHRLDVTEVLQLKVGNAGNPKAQRDAWETDVVSPAARIVGMFLA
VEGARDKDKDRFLLTTPKEVAVPGICTATADWFGQQLDMTVMSPSKGFAGLGSAAAAV
>ZmIPT8
MAAAAVAGARRPLHRTLAHPPPPEEDEHQQQRACRSRGSSSSCSASSSSTPARPRGTGMV
VIVGATGTGKTKLSIDAAEAVGGEVVNADKIQLYAGLDVTTNKVAPADRRGVPHHLLGAI
RPEAGELPPSTFRSLAAATAASIAARGRLPVVAGGSNSLIHALLADRLDAGAADPFSAPPQP
APPRWGRRPALRSPCCLLWVHVDAALLAEYLDRRVDDMVRGGMVEELREYFAATTAAE
RAAHAAGLGRAIGVPELGACFAGRASFRAAIDDIKANTRDLAAAQVRKIRRMADAWGW
PIQRLDASATVRARLRGAGPDAESACWERDVRAPGLAAIRSFLLELDGGSVVDGAVVEEV
EPRVRCCDVVG
>ZmIPT9
MLAPTAAAVSAATATTTLPRPPPSSIDLRRPLERGMALPLQPSLPPPLLLVSANNRHKAVVV
MGATGTGKSRLAVDLALQFGGEVINSDKIQLHAGLEVTTNKVTEEERAGVPHHLLGVAHP
DVEFTAADFRREASRAAGAITARGRLPIIAGGSNSYVEELVDGDGRALREGYDCCFLWVD
VQPAVLHGYVARRVDEMCARGLVDEVAAAFDPRRTDYSRGIWRAIGAPELDAYLRWSGD
EGERARLLAAAIEDIKSNTRRLSCRQRAKIQRLAKLWGVRRVDATEVFRRRGDAADEAW

QRLVATPCIDAVRSFLRDAAVVAAANDPPAAEVVPVFAPASAAVVAG
>ZmIPT10
MSGEMRPQQLGAMRRAIWRSWPILCLQSQHRLLPSFCASTKAATMAASSLPPPTHHKKK
DTVIVISGPTGAGKSRLALEVARRLGGEIISADSVQVYRGLDVGSAKPSAAEMSLVPHHLI
DILDTSDDYSAGAFFRDARRATQDVLDRGCVPVIAGGTGLYLRWYIYGKPNVPQSSMEST
LAVWSELADFRENGQWEEAVELVVQAGDPRARDLSVNNWNRLSRSLEIIRSSGSPPSAFA
LPYDAFCEQHDTDLTEAPSSAGNYEAREMEYDFFCIFLASPRIELYRSIDLRCEEMLVDTGG
LLSEASWLLDIGLQPRMNSASSAIGYKQAMEYLLHCRQNGGESTPQEFLDFLAKFQRTSR
NFAKRQLTWFRNEKIYHWVDGSKPFEALVQFVCDAYHGCSARMVPESLEMKRENCVLKS
RDLKTYRSMNRVFLGGDDCSHVLNWIRRTQSK
>BrIPT1_1
MTELNFLPTISDHFTTTSTSLSFSSHSHFPFSVRMNDQSRPQNLKDKIVVILGATGAGKSRL
SVDIATRLPSEIINSDKIQLYKGLEITTNQITIPDRRGVPHHLLGYLPSEDGELTARGFRIAAS
NAVSDITSRKKLPIIAGGSNTLVHALLAERFDPKIDPFGSSSISRELRYNCCFIWVDVSETVL
FEYLLKRVDDMMGSGMFEELSRFYDPVKASTTRVGIRKAIGVPEFDGYFKMYPPEKEGE
WDSGRRAAYDKVVEEIKENTLRLAKRQVVKIEKLRDAGWDIKRVDATASFRAVLMGRRR
REWRGIWEEQVLEPSVKIVNRLLVED
>BrIPT1_2
MTELNFLPTISDRFTTTSPSPSFSSHSLLPFTHRRTRQSNHRPVSVRMEQSRPRNRKDKIVVI
LGATGAGKSRLSVDLATRFPSEIINSDKIQVYEGLEITTNQITFPDRRGVPHHLLGYLPSEDG
ELTAGDFRADASNAVSDIISRKKLPIIAGGSNSFVHALLAERFDPKIDPFASSSICRDLRYDSC
FIWVDVSEPVLFEYLLKRVDEMMGSGMFEELSGFYDPVKASRARFGIRKAIGVPEFDGYF
KMYPPEKEIKWDSGRRAAYDKAVEDIKENT
LRLARRQVWKIEKLREAGWDIKRVDATASFRAVMMSSSSSREWREIWEEQVLEPSVKIVN
RLLVED
>BrIPT2
MSVNPSNGGIDEAKKKKAKVVVIMGPTGSGKSKLAVDLASHFPVEIINADAMQIYSGLDV
LTNKVTVNEQKGVTHHLLGTVSPDLEFTAKDFRDSTIPLIEEILSRNHVPVLVGGTNYYIQA
LVSKFLLEDSKEDVDECCSNVAPGLDVESISGRDDLSHGYDLLKELDPVAANRVHPNNHR
KINQYLSLHASRGVLPSKLYQENAAENWGCIDASSRFDYCLICMDAETTVLDEYVGQRVD
SMVDAGLLDEVYDIYKPGADYTRGLRQSIGVREFENFLKTYLPDTNTSANDKALKENLR
KILDFPKDDKLRVMLEEAIDSVKLNTRRLLRRQKRRINRLETVFGWNIHHVDATECLLGK
SEESWDVQVVKPATQILQSFLKTETELSHDSTLRKSMERDLWSQYVCEACGNKVLRGRH
EWDHHTQGRVHRKRTARFKKAQNFENREKQEEEEVGICRETS
>BrIPT3_1
MIMRISMAMCKQPLAPSTALNFPPANFGPNMLTLSPSGPKDKVVVIMGATGTGKSRLSVDI
ATRFPAEIINSDKIQVHQGLDIVTNKITTEESCGVPHHLLGVLPPEADLTAVNFRHMANLSV
ESILSRGKLPIIVGGSNSYVEALVDDEDYKFRSRYDCCFLWVDVALPVLHGSVNERVDKM
VANGMVEEVRDFFDYSDSNYSRGIKKAIGVPEFDMFFRNEPFLNTGDREELLSKVVDEIK
RNTFGLACKQRNKIERLRKIKKWCIQRLDATPVFTRRRSKVDADVAWERLVAGPSTDAVS
RFLLDFTSPQPLVEALTAVAREREMSRCLVV
>BrIPT3_2
MIMKISMAMCKQSLPPSTNLEFSPARFGPNMLTLNPYVPKEKVVVIMGATGTGKSRLSVD
LATRFQAEIINSDKIQVHQGLDIVTNKITTEERCGVPHHLLSVLPPQADLTAANFCHMANLS

VESVLNRGKLPIIVGGSNSYVEALVDDDDYKFRSKYDCCFLWVDVALPVLNRFVSERVDK
MVQNGMVEEARDFFDYSDSDYSRGIKKAIGVPEFDIFFRNEPFLNLGDREALLNKVVDEI
KSNTFKLACRQREKIERLRKIKKWCIQRLDATPVITRRRSKVDADVAWERLVARPSTEAVS
RFLLDIDSPRMFVEASTGAVREREMSLVA
>BrIPT5_1
MKPCMTNLRQMIQPMLNFQGISAGLNMVDVPFFRPKDKVVFVMGATGTGKSRLAIDLAT
RFPAEIVNSDK
IQVYKGLDIVTNKVTPEESLGVPHHLLGTVENTHEDFTAEDYQREALRAVKSIVERDRVPII
AGGSNSYIEALVNNCVDFRLRYKCCFLWVDVDRPVLNSFVSDRVDKMVEMGLVDEVRRI
FDPSSSDYSSGIRRAIGVPELDEFLRAEKRDYPAEVTGKLLETAIKKIKENNCLLACRQYQK
IKRLYKQWKWNMHRVDATEVFLRRGEEADEAWESMVARPSALAVDRFLNYSDDHHLEG
ADILLPEISAVPPLPAAVAAISR
>BrIPT5_2
MKPCMTALRQAIPPLLNFKEISTGLNMVDVPFFRRKDKVVFVMGATGTGKSRLSIDLATR
FPAEIVNSDKIQVYKGLDIVTNKVTPEENLGVPHHLLGTVENTHEDFTAEDFQQEAIRAVK
SIVERDRVPIIAGGSNSYIEALVNNCVDFRLRYNCCFLWVDVDKPVLHSFVSERVDKMVE
MGLVDEVRRIFDPTSDDYSTGIRRAIGVPELDEFLRAELLNYPTSKLLETAIKKIKDSNCLL
ASRQYQKIQRLYKQWKWNMHRLDATEVFLRRGEEADDAWEDKVARPSALAVDRFLKYS
EDHHLEGADILLPEISVVPPFPAAVAAISR
>BrIPT7_1
MKFSISAMKQVQPILSFKNKLSMVNVNSFLRPQEKVVFVMGATGSGKSRLAIDLATRFQT
EIINSDKIQVYKGLDVLTNKVTPQECRGVPHHLLGVFDSEAGNLTATDFCRLASQEISTLSA
NNKLPIVAGGSNSYIEALANHSSGFLLNKYQCCFIWVDVSLPVLNSFVSKRVDRMMEAGL
LEEVREVHDPKADYSVGIRRAIGVPELHEYLCYESLVDRGTQRKMLDAAVKKIKENTEIL
ACRQLLKIQRLSKKWKLSMHRVDATEVFLKRNEEEADEAWENLVARQTKRIVDKFCDNQ
VMKNDDVEHCLTSIGAASYGGGSGSRAHNMV
>BrIPT7_2
MKFSISAPKQVQPILSFKNKPSIVNVNSFLHPREKVVFVMGATGSGKSRLAIDLATRFQTEII
NSDKIQVYKGLDVLTNKVTPQECRGVPHHLLGVFDSEDGNLTATDFTLLASQEISTLSANN
KLPIVAGGSNSYIEALANHSSGLLLNNYECCFIWVDVSLPVLNSFVSKRVDRMMEAGLLD
EVRQVYDPKADYSVGIRRAIGVPELHEYLRYESLVDRATQRKMLDVAVKKIKQNTEILAC
RQLQKIQRLSKKWKSCMHHVDATEVFLKHNEEEADEAWENLVARPTKRIVDKFSNRHVM
KNDSVEHCLTTIGAASYGGGSGSRAHNMI
>BrIPT8_1
MQALTSFFVSRSLVPTTPRRPRVRPLSVPPMTVCIDQSRNEKVVVIMGSTGTGKSRLSVDL
ATRFSAEIINSDKMQFYKGLEITTNQITIPERCGVPHHLLGELPVDGPELTASEFRSLASGFIS
EMTSRGKLPIIAGGSNSFIHALLVEHFDPETNPFSKTLIPTELRYDCCFLWVDVSVSVLYHY
LSKRVDQMMESGMFEELADFYNSRDSRSTTRTGIHKSIGVPEFDRYFGVYPPEKNDKVCG
WDPARKVAYEEAVQEIKENTWRLSKKQIDRIMKLRSSGWEIHRLDATASFRAQSREAWDK
NVLEKSVKIVKRFVLEN
>BrIPT8_2
MQELTSTFVSRSLVPTTYTRPPQLRPKSIVPMTVCMDQSRKEKVVVIMGATGTGKSRLSV
GLAARFSGEIINSDKMQFYNGLEIATNQITIPERCGVAHHLLGELPVDDSELTASEFRSVASR
SISEISSRGKLPIIAGGSNSFVHALLVDRFDPVTNPFSSKPSISSELRYDCFFLLVDVSASVLY

HYLSKRVDQMMESGMFEELASFYNPRNSRSTIRTGIHQAIGVPEFDRYFGVYPPEKSHNVF
EWDQARKAAYEEVVHEIKDNTWRLAKKQIERIMMLRSSGWEIHRLDATASLRASSREVW
ENKVLRESVKNVKRFLLKD
>BrIPT9_1
MLSSSSVFLTARTCCLRLQPPCLILRRRLCGATACSVSLNGNTSNKKRNSEKEKVIVISGPT
GAGKSRLALELAKRLNGEIISADSVQVYKGLDVGSAKPSASDRKEVPHHLIDILHPSQDYS
VGQFFEDGRQATKDILSRGRVPIVTGGTGLYLRWFIYGKPDVPKPSPEIISEVHDILLDFQTD
HNWDAAVEFVVNAGDQKASSLARNDWYRLKRSLEILKSTGSPPSSFRVPYDSFRENLNSP
DANDDFSENDISIQNIETDLDYDFLCFFLSSPRIDLYRSIDFRCEDMLSGPNGVLSEARWLL
DLGLLPNTSSATRAIGYRQAMEYLSKCRQQGGVSSPGEFYWFLNKFQQVSRNFAKRQMT
WFRCEPMYHWLNASKPLDTILEFIYDAYEKEAETLVVPDSIRMNKEMRNSREANALKAY
RPRNRHFVGREDCSSVLEWIRNEGCKSDVSCMEGAAI
>BrIPT9_2
MVIIHSGVALTRTCSSRLQPPCLVLRRRFSGAATTCSVSLNGNSEKEKVIVISGPTGAGKSK
LALELAKRLNGEIISADSVQVYKGLDVGSAKPSASDRKEVPHHLIDILHPSQDYSVGQFFE
DGRQATKDILSRGRVPIVTGGTGLYLRWFIYGKPDLPKPSPEIVSEVHDMLIDFQTDYNWD
GAVEFVVNAGDQKASSLPRNDWYRLRRSLEILKSTGSPPSSFRVPYDSFRENLNSHDANN
DFDENGSSADISIQNIETDLDYDFLCFFLSSPRVDLYRSIDFRCEDMLSGANGVLTEARWLL
DLGLLPNTSSATRAIGYRQAMEYLSKCRRQRGVSSPGEFYGFLNKFQQASRNFAKRQMT
WFRCEPMYHWLNASRPLDTILEFIYDAYEKEGETLVVPDSLRMNKDLRNSREANALKGY
RPRNRHFVGREDCSSVLEWISSEGCKSEVSCVEGTTT
>SlIPT1
MRSFFHIHCKNFKFLRNNLCPSYVSYNIATTTVAPLTTTKKIVVIMGATGSGKSKLSIDLAT
RGFNSEIINSDKIQVSKGLDITTNKISINEQSGVVHHLLGEFSGPELFSPSDFRHTADNRITDII
NRRRLPLIVGGSNSFIYALLSNQFNPGVDVFDEINPVQCISKELRYHCCFILVDVLTPVLNRY
LFQRVDEMMNSGMYEELEEFFAKNGFSDRNTGIRKAIGVPEMEGYFRNLKNCTTVQEKC
RLYEAALREIKENTKELAEKQIRKIQRLRESGWDLQKVDATEALRAKMTPGNSKIPATEIW
ERQVVLPSMKIVKQFLLE
>SlIPT2
MRKLSFLSSANHSNTLFFFPKWTRNVVISATSSNVPRLQRSKKIVVIMGATGCGKSKLSIEL
ATHFFPSIEVINSDKIQVYNGLDITTNKISMNERCGIVHHLLGEFKSTESQPEFTPSDFRSAG
DFRINEIVNRGKIPFIVGGSNSFIYALLVKRFDSRFDIFESLNPVSEELRYQCCFIWVDAIAPV
LNQYLDKRVDEMLDSEMFEELKEYFEKEGFSDSGSDGIRKAIGVPEFEKYFKGKISYEEAA
MEIKENTRVLAERQVKKIMRLREGGWNIQRVDATETLTAKMVSEKMAGGENPAGKIWEE
EVLKPSAKIVKQFLLE
>SlIPT3
MNIVLQHIAPFVTCKHQLLINFRDSLTIDPFIPPRRKDKVVIVMGATGTGKSRLSIDLAKQFS
AEVVNSDKMQIYEGLDIVTNKVTDKETCGIPHHLLSIIDPNEVFTATDFCQRASKAVDSIIS
KGQLPIIAGGSNSYIKALVNDDIEFKSRYECCFLWIDVNLKILQKFVSERVDKMVKEGLVD
EAREFFNPDGDYTSGIRRAIGVPEMHQYFINENNKMINENTRDKMLQIAIENIKVNTCKLA
CCQRQNILRLESQLEWKINRLDATEAFEKKDGEAHQAWNRIVYERSTQIVRHFRGEKLLNI
PPPPPPSSSTSFLHKVATMTH
>SlIPT4
MIGMMNSAQMCKQVRWQNLLQLQNFLQKDKVVVVMGVTGAGKSKLSIDLATQFDGEII

NSDKIQVYKGLDIATNKITEEERCGVPHHCLGVIDPYKEFTTKNFCNMASFTVNSITNRGK
LPIIVGGSNSFIEAFVHNSNSYNFSTRYDFCFLWVDASMNVLNSFLYERVDKMVDQGMVD
EVRQIFNPKNMDYTKGIRKAIGVPEFDSYFRAELSNSVDRQTLERMLEEAITEIKINNCILA
SKQLEKIKRLISVKGWKIHRLDASEVFKKQRIAEEKEAEEIWKNMVMGQSRKIVHKFLYE
NYRNSMVYKTDGTAIMAAASHY
>SlIPT5
MEIVEKLQNPNNGGEIKLNPTSNSKPKVVVIMGATGSGKSKLAIDLASHFPIEIINADSMQV
YQGLDVLTNKVTPQEQKGVPHHLLGTISPCVEFTAKDFRDSTISIINEIWLRNRLPVIVGGT
NYYIQALVSPFLLDDSVEDAEANCLSDFQEDEKPDLEVELINEDYDCAYNRLKDIDPVAAN
RIHPNNGRKINQYLNLYARFGVIPSKLLQEKTVEGRTGVKLIIQDMIFASYA
>SlIPT6
MLLGADGILSEARWLLDVGLLPNSNSATRAIGYRQAMEYLLRCREDGGWSSAGDFHDFL
SEFQKESRNFAKRQMTWFRNEQIYEWIDASKPLEKVLSFICDSYNSQDGHLQMPESLRMR
KDIRNHRQAAVLKTYRTINRHFIGHEDCVDVLDWIKKIYGQPTDSLC
>OsIPT1
MEHCNGIAAVGRWLSTKPKVIFVLGATATGKSKLAIRLAARFDGEVINSDKIQAHDGFPVI
TNKVTDEERAGVAHHLLGGVSPDADFTAEDFRREAAVAVARVHAAGRLPVVAGGSNIYVE
ALVAGGGGAFLAAYDCLFLWTDVAPDLLRWYTAARVDDMVRRGLVGEARAGFDAGAD
YTRGVRRAIGLPEMHGYLLAEREGGAGAEDDDDLLAGMLEAAVREIKDNTFRLTVSQVA
KIRRLSALPGWDVRRVDATAVVARMAEGAPHGETWREVVWEPCEEMVSRFLETPAAAAA
VVANGKVDVNVGDAAAGVPEAAAAAVVAAGVV
>OsIPT2
MEYHVGGVIGQSPKPKVVFVLGATATGKSKLAISIAERFGGEVINSDKIQVHDGFPIITNKV
TEEERAGVPHHLLGVLHPDADFTAEDFRREAAAAVARVLAAGRLPVVAGGSNTYVEALV
EGGGGAFRAAHDCLFLWTDVAPGLLRWYTAARVDDMVRRGLVGEARAGFVDGAGAAD
YYTRGVRRAIGIPEMHGYLLAERSGGEAADDGELAAMLDGAVREIKANTYRLAATQVAK
IRRLSALDGWDVRRVDATVVVARMAEGAPHRETWEAVVWKPCEEMVGRFLEASAAVDD
DDNAAAGSPAALAPMTAACRLRAQLVQLQY
>OsIPT3
MEGSRGDKGKVVVVMGATATGKSKLAIDLALRFGGEVINSDKIQVHDGLDVVTNKVTDE
ERAGVPHHLIGGVPPDADYGVDDFRRDAARAVASVLARGRVPVIAGGSNRYLEALLDGE
GGSFRERHELCFLWVDSRAPALHRYVRHRVDRMVEQGLVGEVRGLFRLDDADYSRGIRR
SIGVPEMDAYLRQEATGALLTHGDKYKVALLASAVGEIKANTWSLARRQLRKIHRLRGLP
GWSLRRLDVTRVLELKVEARSEAECAAAWEADVIAPAAREVGMFLHGGGNVVESGREE
QPVVVEKMEVAAVGGAGAAAAAEKWCGRRLLETTAAYHGMEAAAAV
>OsIPT4
MQAYMAVAAAPAPPASLTLLPRTTTVIRDRERFDAAVPVAPLVLRHGAGVKHKAVVVMGA
TGTGKSRLAVDLALRFGGEVINSDKMQIHSGLDVVTNKVTEEECAGVPHHLISVARPDDE
FTAADFRREAARAAAGAVERGRLPIIAGGSNSYVEELVEGDGRAFRERYECCFLWVDVDL
EVLRGFVARRVDEMCRRGLVREVAAAFDPRRTDYSRGIWRAIGVPELDAYLRSRGDGADE
EERARMLAAAVAEIKLNTFRLACRQHRKIERLDRMWRARRVDATEVFRRRGHAADDAW
QRLVAAPCIDAVRSFLFEDQERSSIAAGKPPLFAAGKATSGNISVFASAAAAMAAAAAI
>OsIPT5
MATSLSLAPKPAAVAVAAAAIPRLVPPPSIDMSALSPPPPLVSVSRSMVAKHKAVVVMGATG

TGKTRLAVDLALQFGGEVINADKLQLHRGLDVATNKATADERAGVPHHLIGVAHPDEEFT
AADFRRAASRAAAAVAARGALPIIAGGSNSYIEELVDGDRRAFRDRYDCCFLWVDVQLPV
LHGFVGRRVDDMCGRGMVAEIEAAFDPDRTDYSRGVWRAIGVPELDAYLRSCAAAGGEE
ERARLLANAIEDIKANTRWLSCRQRAKIVRLDRLWRIRRVDATEAFRRRGGAANEAWERH
VAAPSIDTVRSFLHGEFTTAAETTAAPVPPPPFLPMFALAAAGAGV
>OsIPT6
MLNFHPSCDCFLNMLGNKLVVIIGATGTGKTRLSIEIAKAIGGEVVNADKMQIYDGLDITT
NKVSLQDRCGISHHLIASIPRNAGDFPVSFFRSAAKTTINCIARRGHTPIVVGGSNSLIHGLL
VDNFDSSIVDPFGQLEVSYRPTPRSQCCFLWVHVNEVILNEYLKHRVDDMVDAGLVEEIE
EYFDTLSVNGHVPYVGLGKTIGVPELSEYFTGRVSCSDALSMMKTNTQILARSQVTKIHR
MVDVWGWHVHALDCTETILAHLTGSNKYMEDLVWKRDVSDPGLATIQDFL
>OsIPT7
MTSVATRIATLVRAAAAASRPLRLHRRPGGEDTRMVVIVGATGTGKTKLSIDAAKVIGGE
VVNADKIQLYDGLDVTTNKVSLADRRGVPHHLLGAIRPEAGELPPSSFRSLAAATAASIAA
RRLVPVIAGGSNSLIHALLADHFDASAGDPFSPAAAFRHYRPALRFPCCLLWVHVDEALLD
EYLDRRVDDMVDAGMVEELREYFATTTAAERAAHSGLGKAIGVPELGDYFAGRKTFSEAI
DDIKANTRVLAAAQVSKIRRMSDAWGWPIHRLDASDTVRARLTRAGSAAESASWERDVR
GPGLATIRSFLADQSPPPRSEGTNDYLYAMETEPEPPPPPTLPPRLLRLPRMQYCDMVG
>OsIPT8
MERSRVGDGCCCSCSGRGGVASTTAVRPSTGMVVIVGATGTGKTKLSIDAAQELAGEVVN
ADKIQLYDGLDVTTNKVSLADRRGVPHHLLGAIRAEAGELPPSSFRSLAAAAAAGIASRG
RVPVVAGGSNSLIHALLADPIDAAPRDPFADADVGYRPALRFPCCLLWVDVDDDVLDEYL
DRRVDDMVGEGMVEELEEYFATTSASERASHAGLGKAIGVPELGDYFAGRKSLDAAIDEI
KANTRVLAARQVGKIRRMADVWGWPIRRLDATATIRARLSGAGRAAEAAAWERDVRGP
GLAAMRQFVGRADFNAAAVDQLAARSRRQCLRGGMVAG
>OsIPT9
MAHLAASAAPLPSADPDAGEESSHSPPPPEKGLRKVVVVMGATGAGKSRLAVDLASHFA
GVEVVSADSMQVYGGLDVLTNKVPLHEQKGVPHHLLSVIDPSVEFTCRDFRDHAVPIIEGI
LDRGGLPVIVGGTNFYIQALVSPFLFDDMAQDIEGLTLNDHLDEIGLDNDDEAGLYEHLKK
IDPVAAQRIHPNNHRKIKRYLELYESTGALPSDLFQGQATEKWGRPSNSRFDCCFLWLDAD
LHVLDRYVNERVDCMIDDGLLDEVCNIYDREATYTQGLRQAIGVHEFDEFFRFYFARKET
GEIKMDSCTTMAGLHDDNLKGLLDEAVSQLKANTRRLVRRQRRRLHRLNKYFEWNLRHI
DATEAFYGATADSWNMKVVKPCVDIVRDFLSDDTILASRDGSSVTGSPRMSSRELWTQY
VCEACDNRVLRGTHEWEQHKQGRCHRKRVQRLKQKASTVISL
>OsIPT10
MQYGCRRPAVWKRSWSPAAAAATKNKVIVISGPTGAGKTRLALDLAKRLSGEIISADSVQ
VYRGLDVGSAKPSSSDRAAVPHHLIDILHASDDYSAGDFFHDARAATDHLLARARVPIVA
GGTGLYLRWYIYGKPSVPQSSMDVTSAVWSELSRFRDTGRWEEAVDLVANAGDPKARDL
SVNNWSRLRRSLEIIRSSGSPPSAFSLPYNAYNLNHHRRLSLTNQADQPTELELDYDFLCIF
LACPRVELYRSIDLRCEEMLADTGGLLSEASWLLDIGLSPGMNSATCAIGYRQAMEYLLQ
CRHNGGSSSPQEFLEFLTKFQTASRNFSKRQMTWFRNEKIYQWVDASQPFDAIAQFICDAY
HDRAARLVPDSLEMKRESCRHESRDLKTYRSENRVFRGDDDCCHVLDWITRTQRK

>PtIPT2

MESDSAAEEALQNPSNTDGGEKLTVTKKEEKEEKPKLVVIMGPTGSGKSKLAIDLAAHFP
VEIINADSMQVYRGLDVLTNKVPISDQEGVPHHLLGTLNPNVEFTAKDFRDSAIPVLSIGK
CRYLLKCCVVFGIFMVFIIATNNAINGFMLINEILSRNCLPVVVGGTNYYIQALVSPFLLDD
TTNDLDESLLNHPSGDEQTDHATDSGRESFNHSYDYLRELDPVAANRLHPNNHRKINQYL
NLYARSGILPSKLYQGKAAEIFYTRIELCNYFEAVVILEKRMFWQNWGCMDNYRFHCCFIC
VDADIPVLDRYVEQRVDSMIDAGLLGEVCEVYNYNADYTRGLRQAIGVREFDNFLRVYM
SDEKGHDSMGSLFLQSKNEDVKLLKDNMREILHSSDDNQLKILLAEAIDKVKANTRRLVR
VQKRRLTRLQTFFGWNIHYVDATEFISCKSDELWAGQVVSSAVNVIRAFLTEERCAVPDLE
THVGGGMKSVERNLWTQYICKACGNRVLRGAHEWEQHKQGRGHRKRISRLRKSQGHSY
SLVEQEVISNSS
>PtIPT3
MMNLCKSLCPQTSRMLDIPPGMLKMDMLSPRSRQKEKVVIVMGPTGTGKSRLSIELATQF
PAEIINSDKMQVYKGLDIVTNKVAEEEKSGVPHHLLGIANPTVDFTATNYCHTASLAVESIS
TRGLLPIIVGGSNSYIEALMDDEDFRLRLNYDCCFLWVDVSMPVLHKFVSRRVEQMVSVG
MIDEVRNIFDPYADYSTGIRRSIGVPEFDKYFRAEAFLDEENRARLLHEAICHVKKNTCKL
ACRQWEKINRLRKIKGWDIHRLDATEVFQKSGKEAEHAWEMLVARPSTAIVGQLLCGVPA
DKVPAIASVAKNMGYIRQCLVA
>PtIPT5a
MTMRLSLSAYKQVQPRVNFQGGLNMKPFFRRKDKVVFVLGPTGTGKSRLAIDLATHFPA
EVVNCDKMQVYKGLDIVTNKVTEEECRGVPHHLLGIADPNADFTSDDFRHHASLVVESIV
TRDRLPIIAGGSNSYVEALANDDPEFRLRYECCFLWVDVSLPLLHSFVSDRVDRMVRAGLI
DEVRDVFDPTKFDDYSQGIKRAIGVPELDQFLRNETIVDAKTRRKLLDEAIEKIKENTCML
ARRQLQKIRRLHSIWNWKMHRIDATPVFLASGKEADNLWDQIVAGPSTMIVNQFLCEENH
VSPIVPSESINMVPISVPAMAAVASR
>PtIPT5b
MTMRLSLTAYKQVQPRGNFQGGLNMKPFFRRKDKVVFVVGPTGTGKSRLAIDLATRFPA
EVVNCDKMQVYKGLDIVTNKVTEEECRGVPHHLLGIADPNANFTSDDFRHHASLVVESIV
TRDRLPIIAGGSNSYIEALANDDPEFRLRYECCFLWVDVSLPILYSFVSERVDRMVEAGLID
EVRDMFDPNKFDDYSQGIKRAIGVPELDHFLRNEAIVDAKTRRKLLDEAIDKIKENTCML
ASRQLQKIHRLHSIWNWNVHRIDATPVFLTSGKEVDNLWDKLVAGPSTMIVNQFLCDKNY
ASPIIPSESIKMEPISVPALAVGATR
>PtIPT6a
MKIPFPKSTNQPLYTALKTQPLHPINISIPFNKPRPPPIAVRMDTDSSTTTSTAVYRHKKDKIL
VIMGATGCGKTRVSIDLATRFQSEIINSDKMQVYEGLDITTNKITIQDRLGVPHHLLGEFDP
DDGELTPSEYRLAGGLAISGIVSRQNLPIVVGGSNSLIHALVVDRFNPELNVFDGCNPVSTQ
LRYNCCFLWVDVSLPVLCDYLCKRVDEMLDSGMLDELSEYYGSVDAASQIGLRKAIGVP
EFDRYFKKYPPGSGCGRGIGVEWDRVRRGVYEVCVREIKENTCQLAKRQIGKILRLKGAG
WDLKRVDATESFREVMTVTSDDHIKKRKKKRWMEVWGRDVMEPSMKIVKRFLEEE
>PtIPT6b
MVTDSSTAASAGVHHHKKDKILVIMGATGCGKTKVSIDLATRFHSEIINSDKIQVYKGLDII
TNKIPVQDRLGVPHYLLGEFDPEDGELTLSEFRLAGGLAISGIVSRQRLPIVVGGSNSLVHA
LVVDRFNPELNVFDGSNPVSTQLRYNCCFLWVDVSLPVLCDYLCMRVDEMLDSGMFDEL
SEYYGSIDSASQTVLRKAIGGHWWGVGSGTERNIRGVREGDKGEHVSAGKEADWKDLEI
KRCRVGPAKSGCYGEL

>PtIPT7a
MEMIPLQPKLASSMRTMPMAASLPLRMEREIRHRNNLQQITSSLYSIDDKKKQKALFVMG
TTATGKSKLSIDLATHFQGEIINSDKIQVYKGLDMLTNKISEIERRGVPHHLLGFVEPGEEFT
TQDFCNHVHKAMKHITGDGSIPIIAGGSNRYIEALVEDPLFNFKDSYDTCFLWVDVALPILF
DRAAKRVDEMLDAGLVEEVRGMFIPGIDHNSGIWRAIGIAEMEPYFQAEMEMADEVTMK
ILLETGIKEMKENTKKLINKQLTKIKYLANKKGWKFHRIDATCVYERSAKVDEDVWDKK
VLRPSLEIVTNFLREDEKAEEVADSFLVTS
>PtIPT7b
MGTTATGKSKLSIDLATHFQGEIINSDKIQVYKGLDILTNKVSEDESRGVPHHLLGFVEPGE
EFTTQDFCNHVHMAMRHIIGNGNIPIIAGGSNRYIEALVEDPLFKDNYDTCFLWVDVALPIL
FVRAAKRVDKMLDAGLVDEVRGMFIPGIDHNSGIWRAIGIPELEPYFQAEMEMADEVTRK
MLLDTGIKEMKENTKKLINKQLRKIKYLANEKGWKLHRIDATFVYERSGNVDEDVWDD
KVLRPSLEMLTNFLQEDGKAEEFVDASGLPGSFEWREKGVVTDVKIQGACGSCWAFSTT
GSVEGANFIATRKLLNLSEQQLVDCDSVTDKTSCGDGCGGGFMTNAYRCLIEAGALQEET
GLNAILMQTCIRGVSCPVIRGKKWLNRGVLLVGNGARGYSILN
>PtIPT9
MSREQKKGKQEKGGSDVAVKVVVAVKASKEIPKTALVWALTHVVQPGDCITLLVVVPSH
APGRRLWGFPRFAADCANGHRKSHSGATSDQRCDITDSCSQMILQLHDVYDPNKINVKIK
IVSGSPCGAVSAEAKKAQANWVVLDKQLKHEEKRCMEELQCNIVVMKRSQAKVLRLNL
VGTSKEPEVVGPSPSKLNEASEQHSKNKNNSSGSIRGPVVTPTSSPELGTPFTVTEAGTSSV
SSDPGASPFFISETNGELKKEEPLVIKENRDLDESSSDTDTEHLSLASSLRFEPWVGELLGSH
IKSSRHVEESSQRSNCMAQTSTTEALLEKFSKLDRQTGIGMSNYRTDLDLSVNVREAISLS
RNTPPGPPPLCSICQHKAPVFGKPPRWFSYAELELATGGFSQANFLAEGGFGSVHRGVLPD
GQAVAVKQHKLASSQGDVEFCSEVEVLSCAQHRNVVMLIGFCIEDRRRLLVYEYICNGSL
DSHLYGHHREPLEWSARQKIAVGAARGLRYLHEECRVGCIVHRDMRPNNILITHDFEPLV
GDFGLARWQPDGDTGVETRVIGTFGYLAPEYAETGQITEKADVYSFGVVLVELVTGRKAV
DLNRPKGQQCLTEWARPLLEEYAIVELIDPQLGNHYSEQEVYCMLHAASICIRRDPHSRPR
MSQVLRILEGDMHVDTNYMSAPGYDVGNRSGRIYIDRTAAAAAAIKTLWCSAVNE
