# Supplementary material for: Genome-Wide Identification and Expression Analysis of Isopentenyl transferase Family Genes during Development and Resistance to Abiotic Stresses in Tea Plant (Camellia sinensis)
Source: Plants (Basel). 2022 Aug 29;11(17):2243. doi: 10.3390/plants11172243 (PMC9460862; doi:10.3390/plants11172243)
Supplement: Supplementary file 1 [file plants-11-02243-s001.zip › Table S2.pdf]

Table S2 The grand average of hydropathy (GRAVY) and instability index of 13 CsIPT numbers

	GRAVY	instability index
CsIPT2	-0.538	35.46
CsIPT3.1	-0.592	47.67
CsIPT3.2	-0.187	34.29
CsIPT5.1	-0.112	41.68
CsIPT5.2	-0.118	40.54
CsIPT6.1	-0.423	45.51
CsIPT6.2	-0.508	44.41
CsIPT6.3	-0.437	48.57
CsIPT6.4	-0.705	52.07
CsIPT7.1	-0.142	30.15
CsIPT7.2	0.088	25.37
CsIPT9.1	-0.496	42.64
CsIPT9.2	-0.157	43.04
