# Supplementary material for: Genome-Wide Identification and Expression Analysis of Isopentenyl transferase Family Genes during Development and Resistance to Abiotic Stresses in Tea Plant (Camellia sinensis)
Source: Plants (Basel). 2022 Aug 29;11(17):2243. doi: 10.3390/plants11172243 (PMC9460862; doi:10.3390/plants11172243)
Supplement: Supplementary file 1 [file plants-11-02243-s001.zip › Table S3.pdf]

Table S3 *Cis*-elements of the 13 CsIPT promoters

cis-element / Gene name	light responsive	ABA responsive	GA responsive	SA responsive	auxin responsive	MeJA responsive	low-temperature responsive	anaerobic induction	drought inducibility	circadian control	meristem expression
CsIPT2a	7	1	3	1	1	0	0	2	2	0	2
CsIPT2b	8	1	2	1	1	0	0	2	2	1	3
CsIPT3.1	22	12	0	0	0	0	0	4	0	0	0
CsIPT3.2	13	4	0	0	1	0	0	3	0	0	0
CsIPT5.1a	7	1	2	3	0	4	0	2	1	0	0
CsIPT5.1b	14	1	1	3	0	4	0	4	2	0	0
CsIPT5.2a	8	3	1	0	0	10	0	2	2	0	1
CsIPT5.2b	7	0	0	0	0	0	0	2	1	0	0
CsIPT6.1	14	7	0	1	0	8	0	1	0	0	0
CsIPT6.2	8	4	0	0	0	4	0	1	0	0	0
CsIPT6.3	10	0	0	0	0	2	0	0	0	0	1
CsIPT6.4	6	0	1	0	0	6	1	4	2	0	1
CsIPT7.1	14	0	1	0	1	2	0	3	1	1	0
CsIPT7.2	14	3	2	0	0	0	0	5	0	1	0
CsIPT9.1	15	4	0	0	0	0	0	1	2	0	0
CsIPT9.2	17	3	1	0	0	0	0	1	2	0	0
