# Supplementary material for: Genome-Wide Identification and Expression Analysis of Isopentenyl transferase Family Genes during Development and Resistance to Abiotic Stresses in Tea Plant (Camellia sinensis)
Source: Plants (Basel). 2022 Aug 29;11(17):2243. doi: 10.3390/plants11172243 (PMC9460862; doi:10.3390/plants11172243)
Supplement: Supplementary file 1 [file plants-11-02243-s001.zip › Table S4.pdf]

Table S4 The primer sequences used for real-time quantitative PCR (qPCR) of *CsIPT* gene numbers and housekeeping gene of tea plant

Primer	5'→3'	Base number (bp)	PCR product (bp)
CsIPT2-F	TGTGGTTGAACCCTCTGCCA	20	159
CsIPT2-R	AGCACTCGATTGCCACAAGC	20	
CsIPT3.1-F	AAGGTGGTGGTGGTGATGGG	20	86
CsIPT3.1-R	ACTATCTCCGCCGGGAAACG		
CsIPT3.2-F	CTGACGCCTTCCGAAAGCAC		96
CsIPT3.2-R	TGTGAAGGAAGCGGCTCACA		
CsIPT5.1-F	CGGGTTGATCGGATGGTGGA		172
CsIPT5.1-R	GTAGGCGAGCTCTGGTCTGG		
CsIPT5.2-F	CTACGAGCTCGGCTGGGTTT		159
CsIPT5.2-R	CACCACCACCACCTTGTCCT		
CsIPT6.1-F	AATCGACCTCGCAACCCGAT		113
CsIPT6.1-R	ATAGGCGGTCGTGCATTGGA		
CsIPT6.2-F	ATCGACCTCGCAACCCGATT		112
CsIPT6.2-R	ATAGGCGGTCGTGCATTGGA		
CsIPT6.3-F	ACGCCACGGAGGTTATCAGG		69
CsIPT6.3-R	CCCAACTCTCGCCGGAATCT		
CsIPT6.4-F	CAACTGGGTCGGGCAAATCG		102
CsIPT6.4-R	CGAGACCGCGGTAGACTTGT		
CsIPT7.1-F	ACAAGCTTGGTTGGCCGTTG		94
CsIPT7.1-R	GCCACAAGCTCTTCCCATGC		
CsIPT7.2-F	ACTGGTGGAGGACCCTGACT		138
CsIPT7.2-R	GGCCACGACAAACCTGCATC		
CsIPT9.1-F	CTGGGATGCAGCTGTCCAGT		89
CsIPT9.1-R	TGCGACGCAGACGATACCAA		
CsIPT9.2-F	CTCCGGAGCGTAAGGTAGCC		74
CsIPT9.2-R	CAGCTTCATCAGCAGCGCAA		
CsGAPDH F	GAGACTGGAGCCGAATTCATT		166
CsGAPDH R1	GATCTGGCTTGTAATCCTTCTCA		
